# Ethnicity and clinical empathy in primary care consultations: a web-based experiment

**DOI:** 10.3399/BJGPO.2025.0084

**Published:** 2026-03-11

**Authors:** Qingyan He, Rui Du, Saniya Belgi, Greg J Neil, Hazel Everitt, Felicity L Bishop

**Affiliations:** 1 School of Psychology, University of Southampton, Southampton, UK; 2 Department of Psychiatry and Psychology, Guangzhou Women and Children’s Medical Center (National Children's Medical Center for South Central Region), Guangdong, China; 3 Keele Medical School, Keele University, Keele, United Kingdom; 4 Psychology Department, Southampton Solent University, Southampton, United Kingdom; 5 Primary Care Research Centre, University of Southampton, Southampton, UK

**Keywords:** empathy, ethnicity, primary medical care, primary health care, communication

## Abstract

**Background:**

Clinical empathy may enhance patient satisfaction and health outcomes. The interaction between ethnicity and clinical empathy is underexplored.

**Aim:**

To compare how people from different ethnicities perceive GPs' communication of clinical empathy.

**Design & setting:**

A 2 (consultation style) x4 (participant ethnicity) web-based experiment using film vignettes in the UK.

**Method:**

In total, 274 UK Black British, White, East Asian, and South Asian adults (50% female; mean age 39.7 years), recruited from an online participant pool, were randomly assigned to watch one of two films showing enacted GP consultations for osteoarthritis: enhanced consultation (high empathy) or standard consultation (limited empathy). Participants’ perceptions of clinical empathy were assessed quantitatively and qualitatively.

**Results:**

Across the whole sample and within all four ethnicities, enhanced consultations were rated as more empathic than standard consultations; there were no significant effects of participant ethnicity on ratings of empathy. Bayesian analysis confirmed an effect of consultation style and indicated there were no differences between ethnicities in ratings of clinical empathy. Qualitative comments talked about the doctor: (not) showing interest in the patient; responding with(out) respect; (not) conveying warmth, empathy, and hope; and (not) clearly explaining management options or clinical decisions. Participants of all four ethnicities commented on similar aspects of the enhanced and standard consultations.

**Conclusion:**

In this well-controlled experiment, the empathic communication skills modelled in the enhanced consultation were interpreted similarly positively by UK adults of Black British, White, East Asian, and South Asian ethnicities. Core elements of clinical empathy appear relevant and similarly valued across these ethnic groups.

## How this fits in

Studies show there are differences in how people from different ethnicities experience clinician empathy. These differences are incompletely understood and could impact health disparities. This study shows that core elements of clinical empathy appear relevant and similarly valued by UK adults of Black British, White, East Asian, and South Asian ethnicities. If, as part of broader cultural competence, GPs adopted the skills modelled in this study then empathy could be communicated more effectively to UK patients.

## Introduction

Clinical empathy involves healthcare practitioners putting themselves in their patient’s position, acknowledging their patient’s feelings, concerns, and expectations, and communicating that understanding through their behaviour.^1,2^ When healthcare practitioners communicate clinical empathy effectively their patients report higher satisfaction with health care^3^ and, potentially, improved clinical outcomes including reduced pain.^4^ However, much of the evidence for the effects of clinical empathy rests on data from White patients.^5^

Within the broader context of complex ethnic health inequalities in the UK^6^ and globally^7^ the interaction between ethnicity and clinical empathy is potentially very important but remains poorly understood. A systematic review of 40 US studies found that, compared with White patients, Black patients reported poorer communication in general and poorer empathy-related aspects of communication in particular, including positive talk, partnership building, and participatory decision making.^8^ A meta-analytic systematic review of 18 studies found that Black or African American, Asian, and Native Americans perceived their clinician to be less empathetic than White patients and these differences are increasing over time, although are not statistically significant.^9^ However, the primary studies in these reviews typically did not systematically sample patients from diverse ethnic groups, resulting in imbalanced and likely unrepresentative samples. In the UK, patient satisfaction in the GP Patient Survey is consistently lower among minoritised ethnicities; this may be partially attributable to (not) feeling treated with care and concern, a key component of empathy.^10^ In summary, there appear to be differences in how people from different ethnicities experience clinician empathy.^8,9^ Such differences could be explained by clinicians expressing empathy differently to patients of different ethnicities (for example, owing to implicit bias)^11,12^ and/or there might be fundamental differences in how people from different ethnicities perceive similar expressions of clinician empathy during medical consultations.

We aimed to compare how UK adults from different ethnicities perceive GPs’ communication of clinical empathy. The objectives were to test for differences in ratings of clinical empathy between people from four ethnicities: Black British, White, East Asian, and South Asian; examine how any ethnic differences in ratings of clinical empathy interact with the level of clinical empathy shown in a consultation; and explore how participants perceive high and low-empathy consultations.

## Method

### Design

A vignette-based approach tested how the same empathy behaviours performed by the same clinicians were perceived by people from different ethnicities.^13^

A 2×4 between-participants experiment was completed by UK adults. The independent variables were participant ethnicity (four levels: Black British, White, East Asian, South Asian) and consultation style (two levels: enhanced, with high levels of empathy and optimism; standard, with limited empathy and optimism). The dependent variable was participant perceptions of clinician empathy. Covariates were consultation gender (female patient and GP; male patient and GP) and participant gender (female; male).

The experiment was hosted on Qualtrics, a cloud-based survey platform, which enabled us to display participant information sheets and document informed consent; display films to participants; randomly allocate participants to different films; collect responses to closed and open-ended questions; and seamlessly recruit from an online participant pool.

### Participants

Inclusion criteria were UK adults (aged ≥18 years) of Black British, White, East Asian, or South Asian ethnicity. Participants were recruited via Prolific (www.prolific.com) (accessed 12.01.26). Prolific is an online research participant pool providing quick access to a large pool of adults seeking online surveys for payment. Prolific verifies email address, phone number, and photo ID, helping enhance the trustworthiness of this participant pool. Prolific outperforms other crowdsourcing online participant pools in terms of data quality.^14^

We used Prolific’s pre-screening function to invite participant pool members likely to meet our inclusion criteria. Eligibility was confirmed via screening questions on Qualtrics, presented immediately after the participant information sheet and consent. No support was provided for participants with low English proficiency.

Without sufficiently similar work to inform a power calculation, we assumed a medium effect size of ethnicity on perceptions of clinician empathy. To detect a medium effect size (*f* = 0.25) in one-way analysis of variance (ANOVA) with four groups at alpha = 0.05 requires 45 participants per group.^15^

### Materials

#### Measures

Participants reported age (in years), gender (male, female, I define myself in another way, I prefer not to say), ethnicity, highest level of education completed, global subjective health (excellent, very good, good, fair, poor), and whether they had osteoarthritis or cared for someone with osteoarthritis. These items were chosen to facilitate sample description and comparison with the UK population; additional measures of socioeconomic status were not collected to minimise participant burden.

The validated and reliable 10-item Consultation and Relational Empathy (CARE) scale measured participants’ ratings of the GP’s clinical empathy in the filmed consultation.^16^ Each item has six response options: ‘poor’, ‘fair’, ‘good’, ‘very good’, ‘excellent’, and ‘does not apply’; responses were converted to a numerical score and summed to produce an overall rating of clinical empathy ranging from 0 (none) to 50 (high). ‘Does not apply’ responses were replaced with the participant’s average response across completed items. The CARE’s internal consistency was very high in this sample (Cronbach’s alpha = 0.985).

To explore participants’ broader views, two open-ended questions invited participants to share what they liked and disliked about the consultation.

#### Consultation films

Four films were professionally acted and produced for the EMPathicO e-learning package on empathy and optimism for primary care practitioners.^17^ Each film lasted approximately 8 minutes and showed a patient consulting a GP for knee pain, diagnosed in the film as osteoarthritis (a painful and disabling condition commonly managed in primary care;^18^ (see Supplementary Material for transcripts). Four films represented all combinations of consultation style and GP–patient gender: enhanced consultation (high empathy and optimism), male GP, male patient; enhanced consultation, female GP, female patient; standard consultation (limited empathy and optimism), male GP, male patient; standard consultation, female GP, female patient. All actors were White .

### Procedure

In June 2023–July 2023, an advert on Prolific directed potentially eligible individuals to Qualtrics where the participant information statement was displayed with a checkbox to indicate consent. Potential participants then completed screening questions. Eligible participants completed the demographic and health questions, then were randomly allocated to watch one of the four consultation films (stratified by participant ethnicity). After watching one film, participants completed the empathy items and reported any interruptions, technical or other difficulties. Participants were thanked and redirected to Prolific for payment.

### Analysis methods

Self-reported ethnicity, gender, and age collected via Qualtrics were compared with responders’ prolific profiles; any with discrepancies were excluded. Responders were excluded if they reported stopping watching before the film ended (*n* = 1), were interrupted while watching (*n* = 11), or completed the study quicker than film duration (*n* = 5). A factorial ANOVA tested for (fixed) effects of consultation style and participant ethnicity on CARE scores. Including participant and actor gender as covariates did not alter the inferences. Inspection of Q-Q plot and histogram confirmed residuals approximately followed the Normal distribution. Effect sizes indicated by partial eta-squared are interpreted according to Cohen.^19,20^

In traditional hypothesis testing, a non-significant effect (for example, of factor x) on a measure (for example, measure y) means there is no evidence of an effect of factor x on measure y. This is not the same as evidence that there is no effect of factor x on measure y. Rather, it could mean either that the study was insufficiently powered to detect an effect or that there is no effect to be detected. Traditional hypothesis testing cannot distinguish between these explanations, but Bayesian statistics can supplement traditional approaches by generating evidence that there is no effect of factor x on measure y.^21^ Table 1 summarises this approach to null effects.^22,23^

**Table 1.** Introductory overview of Bayesian approach to null effects

Feature	Description
Approach	Bayesian analyses involve testing whether observed data are more or less likely to occur under different models. For example, we could compare the likelihood of the observed data occurring under a model with no effect of x on y (the null model), to the likelihood of the observed data occurring under a model with an effect of x on y (the main effect model).
Statistic	The Bayes factor (BF_01_) indicates the relative likelihood of the observed data occurring under one model compared with the other model. If BF_01_ for the null model compared with the main effect model is greater than 1, then the observed data are more likely to be generated by the null model, and we conclude that there is evidence that x does not have an effect on y.^22^
Interpretation	Unlike *P*-values, Bayes factors cannot be assigned significance but their magnitude does indicate strength of evidence: <1 BF_01_ <3 indicates anecdotal evidence; <3 BF_01_ <10 moderate evidence; <10 BF_01_ <30 strong evidence; <30 BF_01_ <100 very strong evidence; <100 BF_01_ extreme evidence.^23^

Following a non-significant effect in the traditional ANOVA, we conducted a 2×4 Bayesian ANOVA using JASP (version 0.19.3). We used the default prior option of 0.5 for the fixed effects; this is an appropriate approach to modelling when there are no strong (empirical) grounds for choosing other priors. Sensitivity analyses run with priors of 1 and 0.2 did not change the conclusions.

Qualitative data were read repeatedly before being classified as positive or negative about the consultation, and then further categorised into inductive, data-driven categories. Most comments were very brief and many mentioned multiple aspects of the consultation (for example, body language and advice); categorisation was not mutually exclusive (that is, one comment could be placed in multiple categories). Categories were then mapped to participant ethnicity and comments were compared qualitatively. NVivo (version 12) was used to facilitate categorisation and to compare the nature of comments made by people from different ethnicities. The qualitative analysis was led by RD and FB, who discussed the categorisations in depth. Quotations for this report were selected to clearly illustrate the categories and are attributed to individuals using participant numbers.

## Results

### Participants

There were 274 participants, aged between 18 years and 77 years (mean [M] = 39.7, standard deviation [SD] = 14.2). A slightly higher proportion of participants reported being White (32%) than Black British (23%), East Asian (20%), or South Asian (25%) (Table 2). The gender split overall was balanced between males and females; this was also the case for three ethnicities, but there were more males than females among South Asian participants (60% versus 40%). Overall, most participants across all ethnic groups reported having obtained a university degree. Most participants reported having good (36%) or very good (34%) general health; self-reported health was broadly similar across ethnic groups. Fewer than 10% of the whole sample had osteoarthritis themselves or cared for someone with osteoarthritis.

**Table 2.** Participant characteristics

	Whole sample (*n* = 274)		Black British (*n* = 63)		White (*n* = 88)		East Asian (*n* = 55)		South Asian (*n* = 68)	
	*n*	*%*	*n*	%	*n*	%	*n*	%	*n*	%
**Gender**										
Male	136	49.6	28	44.4	43	48.9	24	43.6	41	60.3
Female	137	50.0	35	55.6	44	50.0	31	56.4	27	39.7
Non-binary	1	0.4	0	0	1	1.1	0	0	0	0
**Highest education**										
No formal education qualification	1	0.4	0	0.0	1	1.1	0	0	0	0
GCSEs or O levels or similar	23	8.4	3	4.8	17	19.3	1	1.8	2	2.9
A levels or similar	48	17.5	12	19.0	22	25.0	3	5.5	11	16.2
Degree or similar	124	45.3	24	38.1	30	34.1	27	49.1	43	63.2
Postgraduate degree	75	27.4	23	36.5	16	18.2	24	43.6	12	17.6
Other	3	1.1	1	1.6	2	2.3	0	0.0	0	0
**Self-rated health**										
Excellent	33	12.0	13	20.6	4	4.5	4	7.3	12	17.6
Very good	93	33.9	20	31.7	27	30.7	24	43.6	22	32.4
Good	99	36.1	26	41.3	30	34.1	18	32.7	25	36.8
Fair	40	14.6	3	4.8	22	25.0	7	12.7	8	11.8
Poor	9	3.3	1	1.6	5	5.7	2	3.6	1	1.5
**Osteoarthritis experience**										
Has osteoarthritis	16	5.8	2	3.2	12	13.6	0	0.0	2	2.9
Is a carer for someone with osteoarthritis	21	7.7	4	6.3	3	3.4	6	10.9	8	11.8

### Effects of ethnicity on ratings of empathy

Across the whole sample and within all ethnic groups, enhanced consultations were rated as more empathic than standard consultations (Table 3). The traditional factorial ANOVA confirmed a large and statistically significant main effect of consultation style on ratings of clinical empathy, F(1,266) = 814.81, *P*<0.001, partial η^2^ = 0 .75. There was no main effect of participant ethnicity on ratings of clinical empathy, F(3,266) = 2.039, *P* = 0.109, partial η^2^ = 0.02. While there appeared to be greater variation between ethnic groups in ratings of the standard consultation compared with ratings of the enhanced consultation (Table 3), the interaction between consultation style and participant ethnicity was small and not statistically significant, F(3,266) = 2.087, *P* = 0.102, partial η^2^ = 0.02.

**Table 3.** Mean CARE scores for standard and enhanced consultations by ethnicity

	Standard consultation			Enhanced consultation		
Participant ethnicity	Mean	SD	*n*	Mean	SD	*n*
Black British	19.09	10.52	33	44.90	5.13	30
White	16.14	5.44	44	44.93	5.27	44
East Asian	22.14	9.80	29	44.62	4.73	26
South Asian	19.46	10.85	35	46.12	4.23	33
All	18.89	9.32	141	45.16	4.87	133

CARE scores possible range 10–50; higher scores indicate greater perceived clinical empathy. CARE = Consultation and Relational Empathy

In the Bayesian analysis, all possible models were assessed and compared with the best-performing model (Table 4). The comparison between the model that included only consultation style and the null model (with no predictors) overwhelmingly favoured the consultation-style only model, BF_01_ 1.784×10^+81^; this confirms the results of the traditional ANOVA that there was an effect of consultation style on perceptions of empathy. The comparisons between the consultation style-only model and models that included consultation style and participant ethnicity also favoured the consultation style-only model. The data were 8.53 times more likely to be observed under the consultation style-only model than under the model with main effects of consultation style and ethnicity and their interaction. Furthermore, the data were 4.05 times more likely to be observed under the consultation style-only model than under the model with main effects of consultation style and ethnicity. These comparisons constitute moderate evidence in favour of the consultation style-only model compared with the models also including participant ethnicity,^23^ that is, there is moderate evidence that there is no effect of participant ethnicity on perceptions of clinical empathy.

**Table 4.** Bayesian analysis model comparison

Model (M)	P(M)	P(M|data)	BF_M_	BF_01_	Error %
C	0.20	0.733	10.990	1.000	
*C* + E	0.20	0.181	0.883	4.05	0.990
*C* + E + C X E	0.20	0.086	0.376	8.53	1.390
Null model	0.20	4.109×10^–82^	1.643×10^–81^	1.784×10^+81^	1.994×10^–85^
E	0.20	1.071×10^–83^	4.285×10^–83^	6.844×10^+82^	0.012

*C *= consultation style. *E *= participant ethnicity. C X *E *= the Interaction between C and E. Model (M) the variables included in each model. P(M) The prior model probability. P(M|data) Posterior model probability. BF_M_ the posterior model odds. BF_01_ represents the Bayes factors, which indicates how much more likely the best model is (the model on the top row) compared with the model on the current row; for instance, the data is 8.53 times more likely under the model with just consultation style, compared with the model with both main effects and an interaction (third row from the top). Error % error in computation of the Bayes factor.

To further illustrate this, an analysis of effects was used. This gives the Bayes factor for whether consultation style, participant ethnicity, and the interaction, should each be included or excluded from the final model. There was overwhelming evidence that consultation style should be included in the model (BF_incl_ >100), ‘anecdotal’ evidence that the interaction should not be included in the model (BF_incl_ = 0.38), and moderate evidence that participant ethnicity should not be included in the model (BF_incl_ = 0.24). Overall, the findings of the Bayesian analysis support the traditional ANOVA and add that the evidence strongly favours this interpretation: there was only an effect of consultation style, and that there were no differences in ratings of clinical empathy owing to participant ethnicity.

### Participants’ qualitative comments about the consultations

Two hundred and nine participants (76%) commented on either positive (89, 32% of all participants), negative (54, 20%), or both positive and negative (66, 24%) aspects of the consultation they watched. Enhanced consultations received more positive than negative comments (96 versus 20), standard consultations received more negative than positive comments (100 versus 59); this pattern was consistent across participants of Black British, White, East Asian, and South Asian ethnicities.

Comments were categorised into talk about the doctor: (not) showing interest in the patient; responding with(out) respect; (not) conveying warmth, empathy, and hope; and (not) clearly explaining management options or clinical decisions (see Table 5). In addition to commenting on aspects of the consultations that they liked and disliked, some participants related the enacted consultations to real-life experiences (see Table 6). All categories included comments about similar issues by participants from all four ethnicities. In other words, there were no major differences in how participants from different ethnicities talked about the consultations.

**Table 5.** Categorised comments about consultations

Category name and description	Illustrative positive comments	Illustrative negative comments
(**Not) showing an interest in the patient**		
Participants commented on how familiar the doctor was with the patient’s case, how interested they seemed to be in the patient as a person, and how focused and attentive they appeared to be when the patient was talking. Positive comments mentioned the doctor’s careful questioning, attentive listening, and open posture. Negative comments mentioned the doctor interrupting or rushing the patient, repeating their questions, seeming distracted, or focusing on their computer.	*‘I really liked how the doctor treated the patient with full attention and how he listened to what the patient had to say. Doctors really give you that much time or listen to you with that much patience, that’s the most important thing and that’s what I look for when I am looking for a doctor.’* (P182) *‘I liked that she was very good at asking follow-up questions.’* (P135)	*‘He* [the male doctor] *seemed disinterested, didn’t appear to listen to the patient carefully as he kept asking questions that the patient has already answered, kept interrupting the patient.’* (P319) *‘Poor demeanour, body posture (arms folded, sitting back). Not paying attention (typing away at pc).’* (P303)
**Responding with(out) respect**		
Participants commented on how the doctor addressed, dismissed, or ignored the patient’s concerns. Negative comments mentioned the use of medical jargon or acronyms and other forms of language that were not tailored to their patient. Positive comments described the doctor as being honest, respectful, or non-judgemental.	*‘The doctor addressed the concerns that the patient brought up clearly, it seems like they answered the questions the patient have.’* (P159) *‘Paying attention and allowing the patient to tell her story and proffering possible solution for the patient to try. suggesting ibuprofen gel since the tablet/capsule makes the patient feel sick.*’ (P50)	*‘*[The female doctor] *didn’t listen to the patient when she mentioned her concerns regarding the amount of tablets she had to take — just continued saying “if it works, it’s alright really“. Mentioned NSAIDS and TKR* [total knee replacement]*- medical jargon that she didn’t bother to explain to the patient. Didn’t address the patient’s concern that she was back in the same place as she was 6 months ago.’* (P381)
**(Not) conveying warmth, empathy, and hope**		
Participants liked it when the doctor built rapport with the patient and came across as warm, friendly, sympathetic, considerate, reassuring, empathetic, and/or caring, and offering some positivity or hope to the patient. Negative comments described the doctor as cold and uncaring, as being without compassion, empathy, sympathy, or warmth, and as being pessimistic or not fostering hope in the patient.	*‘I liked every bit of what the doctor represents. She was so kind and compassion yet so professional at the same time.’* (P73) *‘I liked the doctor’s tone of voice and speaking speed. It was very reassuring and made the patient comfortable.’*	*‘Seemed cold and indifferent.*’ (P237) *‘She* [the doctor] *just seemed to suggest some way of compromising, all the while emphasising that it might not work. The patient seems to feel very helpless.’ (P318)*
(**Not) clearly explaining management options or clinical decisions**		
Participants liked it when the doctor involved the patient in decision making, and provided clear and concise descriptions of possible options, explanations for clinical decisions, and recommended next steps. Participants did not like it when they thought the doctor did not explain why they did not recommend the patient’s preferred option (in this scenario, surgery).	‘*He made it very clear to the patient what he needed to do and what would help.*’ (P139) *‘The doctor carrying the patient along in the decision making was the highlight of the video for me, I really liked that.’* (P55)	*‘Good that at least he wrote the medication regime down for the patient — but I wanted to hear a — right — this is what we're going to do to help 3-point plan covering 1. medication increase; 2 exercise increase; 3 wt loss. Needed to explain why a TKR wasn't appropriate for him at this stage.’* (P270)

**Table 6.** Selected quotes illustrating participants relating filmed consultations to personal experiences

	Standard consultation	Enhanced consultation
How filmed consultation relates to personal or vicarious experience	‘*Not good in my opinion but sadly very familiar. My wife has been through such like appointment and it’s very frustrating.’* (P245)	‘*Wish my doctor was as understanding and spent as much time talking through issues. Example is very different to my experience!’* (P131)
What if participant were to experience filmed consultation	*‘I would be very disappointed if I had such a consultation with a GP who was like this.’* (P267)	*‘I liked it all! I wish all GP consultations were like the one I just viewed.’* (P74)

## Discussion

### Summary

Participants from all four ethnicities included in the study perceived the enhanced consultations as significantly more empathic than the standard consultations (*P*<0.001). Bayesian analysis confirmed the effect of consultation style and further indicated that there was no effect of ethnicity on ratings of empathy. Qualitative comments were markedly more positive and less negative about the enhanced consultations.

### Strengths and limitations

Participants rated enacted consultations; they were observers rather than personally experiencing a clinical interaction. Only a small proportion shared the health concerns of the patients in the films and this lack of personal relevance may have blunted their responses, although this is unlikely given the extreme scores on the CARE scale and strongly worded comments. Providing videos in this experimental design meant controlling for variables, including the patient’s clinical condition and individual GP variability, which would not be possible in a clinical setting. Thus, we traded ecological for internal validity. The actors in the filmed consultations were all White; this may have shaped participants’ views in unknown ways. We successfully recruited similar numbers of participants across four ethnicities and the gender split within ethnicities broadly reflects 2021 census data, except for South Asians where our sample contained more males than the census (60% versus 50%, respectively). However, our participants were more highly educated than the general population: only 0.4% of participants had no formal qualifications, compared with 18% of adults in the 2021 census^24^ and likely had high English language proficiency.

Future studies could consider the following: recruiting participants with no formal educational qualifications from more deprived populations with higher levels of morbidity, as deprivation and multimorbidity can impact perceptions of clinical empathy;^25,26^ examining the impact of English language proficiency on perceptions of clinical empathy across ethnicities, given documented impacts of English language proficiency on patient–practitioner communication;^27^ exploring whether and how intersectional positions (for example, different combinations of gender and ethnicity for patients and clinicians) might shape experiences of clinician empathy;^28^ including participants from other ethnic groups or using more precise ethnic categorisations (for example, Arab, Bangladeshi, Chinese, White Irish);^29^ and increasing the sample size to permit detection of small effects of ethnicity on ratings of clinician empathy and interactions between ethnicity and consultation style. A qualitative approach, for example, using the enacted consultations to stimulate focus group discussions, would potentially elicit more nuanced data.

### Comparison with existing literature

Our findings suggest that relatively highly educated UK adults of Black, White, South and East Asian ethnicities with good self-reported health perceive similar levels of clinician empathy from the same clinician behaviours. This rules out one possible explanation for understanding the documented ethnic disparities in satisfaction with real-world communication and empathy in primary care.^10,28^ Specifically, our findings support the need for further research to explore whether some clinicians communicate differently to patients depending on patient ethnicity, potentially owing to implicit biases, and whether intersectional factors, such as educational level, multimorbidity and socioeconomic factors, are also important. Addressing implicit biases may help reduce communication disparities^11,12^ but should be complemented by relevant accessible practitioner training to enhance empathy skills and embed them in all consultations.

### Implications for practice

It is widely acknowledged that not all clinical consultations have optimal communication. If as part of broader cultural competence, clinicians are enabled to adopt the skills modelled in the enhanced consultation film (and taught in the broader EMPathicO intervention),^17^ empathy could be communicated more effectively to UK patients of Black British, White, East Asian, and South Asian ethnicities.

## Data

The anonymised data that support the findings of this study are available in eprints http://eprints.soton.ac.uk at https://doi.org/10.5258/SOTON/D3611.
